# Is the Health Behavior in School-Aged Survey Questionnaire Reliable and Valid in Assessing Physical Activity and Sedentary Behavior in Young Populations? A Systematic Review

**DOI:** 10.3389/fpubh.2022.729641

**Published:** 2022-03-28

**Authors:** Yang Su, Yanjie Zhang, Si-Tong Chen, Jin-Tao Hong, Hongying Wang

**Affiliations:** ^1^School of Physical Education and Sport Training, Shanghai University of Sport, Shanghai, China; ^2^School of Physical Education and Humanity, Nanjing Sport Institute, Nanjing, China; ^3^Physical Education Unit, School of Humanities and Social Science, The Chinese University of Hong Kong–Shenzhen, Shenzhen, China; ^4^Institute for Health and Sport, Victoria University, Melbourne, VIC, Australia; ^5^Center of Physical Fitness Research and Health Guidance, Shanghai Research Institute of Sports Science (Shanghai Anti-doping Agency), Shanghai, China; ^6^School of Leisure Sport, Shanghai University of Sport, Shanghai, China

**Keywords:** behavioral epidemiology, physical activity, sedentary behavior, measurement, children and adolescents, health behavior in school-aged children

## Abstract

**Backgrounds:**

Using the self-reported questionnaire to assess the levels of physical activity (PA) and sedentary behavior (SB) has been a widely recognized method in public health and epidemiology research fields. The selected items of the Health Behavior in School-aged (HBSC) Survey Questionnaire have been used globally for measurements and assessments in PA and SB of children and adolescents. However, there are no comprehensive and critical reviews to assess the quality of studies on reliability and validity of selected items for PA and SB measurement and assessment derived from the HBSC. Thus, this review aimed to critically assess the quality of those studies and summary evidence for future recommendations.

**Methods:**

A systematic review protocol was used to search potentially eligible studies on assessing reliability and validity of PA and SB measures of the HBSC questionnaire. electronically academic databases were used. The information on the reliability and validity of the PA and SB measures were extracted and evaluated with well-recognized criteria or assessment tools.

**Results:**

After a literature search, six studies were included in this review. The reliability of PA measures of the HBSC questionnaire showed a moderate agreement while the reliability of SB measures showed a great variation across the different items in the different subgroups. The validity of the PA measures had acceptable performance, whereas no studies assess the validity of the SB measures. The included studies all had quality weaknesses on reliability or validity analysis.

**Conclusions:**

The PA and SB measures of the HBSC questionnaires were reliable in assessing PA and SB among adolescents. However, a little evidence showed that PA measures are partially valid in assessing PA, but no evidence confirmed the validity of SB measures. The included studies all had methodological weaknesses in examining the reliability and validity of the PA and SB measures, which should be addressed in the future. Further studies are encouraged to use a more standardized study design to examine the reliability and validity of the PA and SB measures in more young populations.

## Introduction

It is well-known that sufficient physical activity (PA) and limited sedentary behavior have been two key determinants of health outcomes among children and adolescents, such as improved fitness, reduced body fat, increased cognitive ability, lower levels of depression and anxiety as well as fewer suicidal attempts (1–8). The World Health Organization (WHO) and some national health sectors have released the guidelines on PA and SB based on epidemiological evidence, which recommend that children and adolescents should amass at least of 1 h for moderate to vigorous PA and <2 h of SB during leisure time (9, 10). Despite numerous health benefits resulting from PA and SB based on convincing evidence, the prevalence of meeting the PA and SB guidelines was not ideal. Specifically, a global study including 1.6 million participants by Guthold et al. (11) reported that only about 20% of adolescents were physically active according to the PA guidelines. This result was highly similar to another study published in the Lancet 2012 PA Research Series (12). In the face of this concerning public health issue, it is of vital significance to promote PA while discouraging SB concurrently among children and adolescents (13).

To increase PA and decrease SB, an essential step is to know and understand the actual levels of PA and SB (e.g., prevalence of meeting the PA or SB guidelines, or time spent in PA or SB) accurately (12, 14–16). At a populational level, using self-reported questionnaires to collect data or information on PA and SB is a feasible and economical measurement because of its lower costs, reduced testing burdens, and easy data management (17–20). To date, there are many specific questionnaires to assess PA and SB level, such as the International Physical Activity Questionnaire (IPAQ), the Global Physical Activity Questionnaire (GPAQ) (21), the Health Behavior in School-aged Questionnaire (HBSC) (22). These questionnaires have been used frequently across the populations in different countries (23–30). Among the three questionnaires, the HBSC questionnaire is typically designed for assessing child and adolescent health behaviors, including PA and SB. In the HBSC questionnaire, some selected items are used for PA and SB measurement, of which four items were used for PA measurement and eight items were used for SB measurement. Using the measures from the HBSC questionnaire for PA and SB (selected items), many national estimates, reports, or studies of levels in PA and SB at the young population level have been published previously (31–33), which in turn provide national comparable evidence.

Although the PA and SB measures derived from the HBSC questionnaire has been tested for reliability and validity in multiple young populations (e.g., Chinese, Japanese, and Slovakian) (34–36), no systematic review studies assess those studies comprehensively and summarize evidence on reliability and validity of the PA and SB measures. This would be a barrier to making an overview of the studies using the PA and SB measures of the HBSC questionnaire. Also, being unaware of the quality of these studies is indeed a critical question for further behavioral epidemiological research and populational monitoring and surveillance. Another issue on this research topic is that there are no studies to assess the quality of studies on reliability and validity of the PA and SB measures. If researchers understand the information on reliability and validity, it would be beneficial to understand the assessed PA and SB levels among the young population through the HBSC questionnaire.

Thus, this review aimed (1) to comprehensively assess the studies on reliability (test-retest) and validity (criterion) of PA and SB measures derived from the HBSC questionnaire; (2) to evaluate the testing performance of PA and SB measurements of the HBSC questionnaire. It could be expected that this review can provide valued and supportive information for future studies using the HBSC questionnaire to assess PA and SB, and then offer implications for future research recommendations.

## Methods

### Literature Search

To achieve the research aims of this study and followed PRISMA guidance, 6 electronic databases were used to perform the literature search, including EBSCO (Full), Elsevier, Medline, PubMed, SPORTDiscus, and Web of Science. The following search terms were used: (1) measure^*^(i.e., measures, measurement), assess^*^(i.e., assessed, assessment), (2) reliab^*^ (i.e., reliable, reliability), (3) valid^*^(i.e., valid, validation, validity), (4) accura^*^(i.e., accurate, accuracy), (5) observ^*^ (i.e., observed, observation), (6) propert^*^(i.e., property, properties), (7) consistency, (8) agreement, and (9) health behavior in school-aged children as well as 10) HBSC. The literature search was done on 31 December 2020, by two authors (SC and JH).

### Selection Criteria

**Figure 1:**
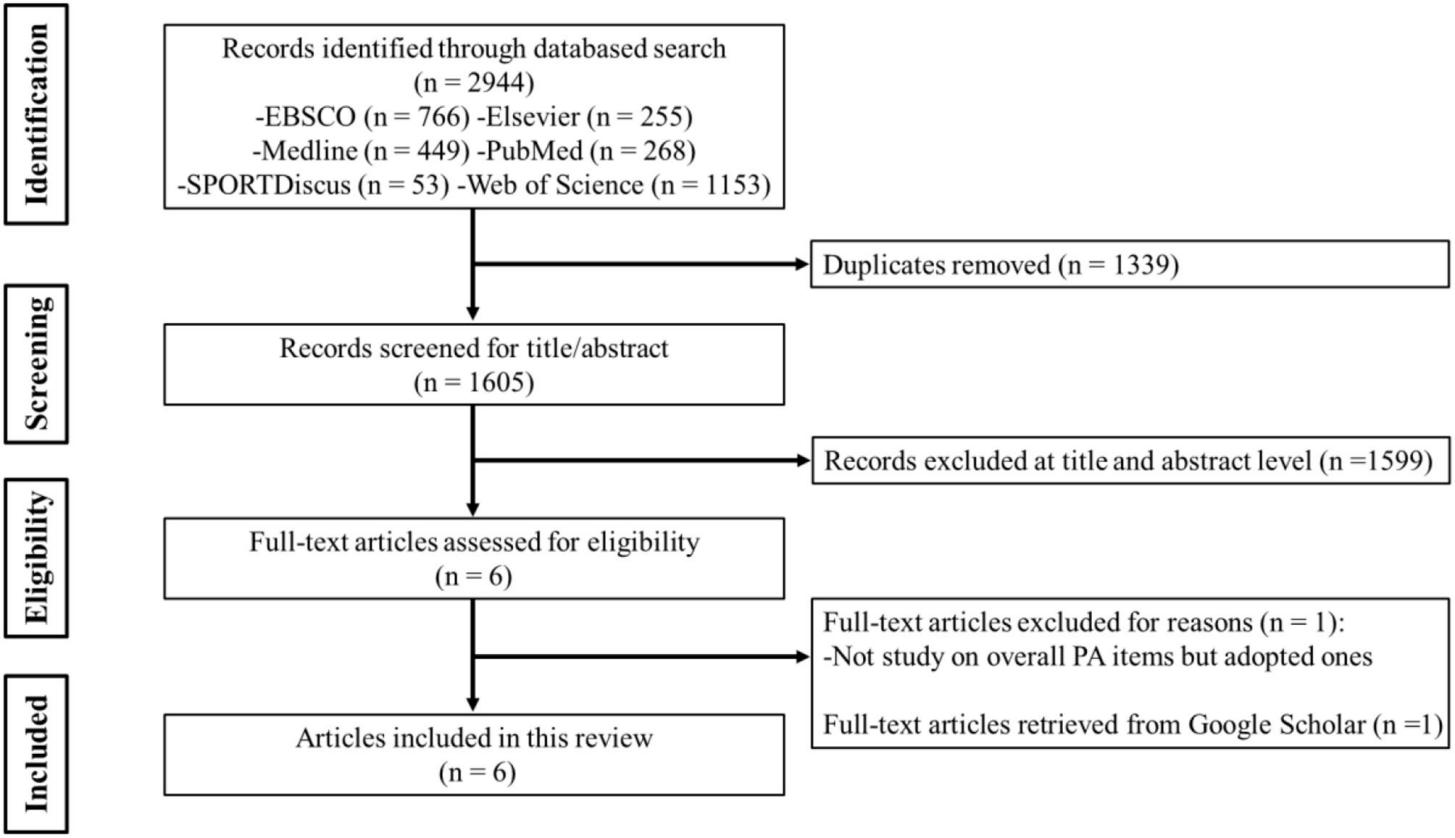
Flow chart of literature selection.

Papers based on the searches were screened against the following inclusion criteria: (1) full-text original report published in a peer-reviewed journal; (2) the study participants were healthy or typically developed; (3) the study participants were children or adolescents; (4) the study that reported either reliability or validity information of PA or SB measurement; (5) published language is English. The exclusion criteria for study selections detailed: (1) studies published as a conference paper, review, or meta-analysis; (2) studies published not in English; (3) studies not using measures for PA and SB from the HBSC questionnaire. Finally, following the literature search protocol and study screening process (see Figure 1), 6 eligible studies (34–39) meeting the literature selection criteria were included in this review.

### Data Extraction

Information was extracted from the included studies regarding the first author, published year, sample characteristics (e.g., sample size, % of sex), PA and SB measures (questions of PA and SB measures), statistical analyses, information on reliability (e.g., intraclass correlation coefficient, ICC; interval days) or validity (e.g., criteria validity correlation coefficient; objective standard). Two independent reviewers (YS and YZ) conducted data extraction, and any disagreement of them was discussed with and resolved by a third author (HW). If some studies reported the information on reliability and validity by age (grade) groups, sex, or other sociodemographic factors, those results were also extracted. The extracted data from the included studies are shown in tabular format.

### Methodological Quality Assessment of the Included Studies

Using the consensus-based standards for the selection of health measurement instruments (COSMIN) (40), the included studies were rated. This checklist was used for the assessment of the methodological quality of the included six studies. Two authors (YS and YZ) of the review independently conducted the quality assessment; any differences between the independent assessments were resolved through discussion between the third author (HW) until they reached an agreement. For test-retest reliability, 10 mandatory items involved study design for quality assessment, and 4 optional items involved depended on each the statistical analysis of each study (some studies used ICC while others used Cohen's kappa to assess the reliability). Hence, the full score the of test-retest reliability analysis of each study were not the same (11–14 scores). For criterion validity, five mandatory items involved study design for quality assessment and two optional items involved depended on each study's statistical analysis. Hence, each study's full score of validity analysis varied (6 or 7 scores).

For the results of reliability and validity (coefficients), the criterion developed recommended by Landis and Koch (41) was used to assess the performance of reliability and validity of each included study. This criterion has been used frequently across the previously published studies (42–46). In detail, coefficient values of <0.2 were considered poor, 0.21–0.4 were considered fair, 0.41–0.6 were regarded as moderate, 0.61–0.8 were deemed substantial, and 0.81–1.0 was almost perfect.

## Results

Table 1 summarizes the specific questions or items for PA and SB measures derived from the HBSC questionnaire. In detail, PA measures of the HBSC questionnaire cover four indicators of PA, including moderate to vigorous PA (MVPA) over the past week or over the usual week, frequency of vigorous PA (VPA), and duration of VPA. Concerning SB measures of the HBSC questionnaire, TV time, computer time, and sitting time are three main domains of assessed SB. The responses to each question or item are shown in Table 1 as well.

**Table 1:** Specific questions and their responses of each included study used as physical activity and sedentary behavior in this review.

**HBSC measures**	**Questions**	**Measured domains**	**Responses**
Physical activity	Over the past 7 days, on how many days were you physically active for a total of at least 60 min per day?	Moderate to vigorous physical activity over the past week (past week MVPA)	0 days; 1; 2; 3; 4; 5; 6; 7 days
	Over a typical or usual week, on how many days are you physically active for a total of at least 60 min per day?	Moderate to vigorous physical activity over the usual week (typical week MVPA)	0 days; 1; 2; 3; 4; 5; 6; 7 days
	Outside school hours: How often do you usually exercise in your free time so much that you get out of breath or sweat?	Frequency of vigorous physical activity (VPA frequency)	Daily; 4–6 times a week; 2–3 times a week; Once a week; Once a month; Less than once a month; Never
	Outside school class: How many hours a week do you usually take physical exercise in your free time so that you lose your breath or sweat?	Duration of vigorous physical activity (VPA duration)	None; About. half an hour; About. an hour; About. 2–3 h; About 4–6 h; 7 hours or more
Sedentary behavior	How many hours a day, in your free time, do you usually spend watching TV, videos (including YouTube or similar services), DVDs, and other entertainment on a screen on weekday and weekend days, respectively?	TV time	None at all/About half an hour a day/About 1 h a day/About 2 h a day/About 3 h a day/About 4 h a day/About 5 h a day/About 6 h a day/About 7 or more hours a day
	How many hours a day, in your free time, do you usually spend using electronic devices such computers, tablets (like iPad), or smartphones for other purposes, for example, homework, emailing, tweeting, Facebook, chatting, surfing the internet on weekday and weekend days, respectively?	Computer time	
	Outside school hours: How many hours a day do you usually spend time sitting in your free time (for example, watching TV, using a computer or mobile phone, traveling in a car or by bus, sitting and talking, eating, studying)?	Sitting time	

*HBSC, Health Behavior in School-aged Children; TV, television*.

Table 2 presents specific information of the included study in this systematic review. The published year of the included studies ranged from 2001 (39) to 2019 (37), with an interval of 18 years. The sample size of the included studies ranged from 70 (36) to 2,752 (37). The six included studies were conducted in Finland (37), Japan (36), Slovakia (35), Czechia (35), Poland (35), China (34), Norway (38), and Australia (39). Most studies targeted study participants as adolescents aged 11–15 years. Five studies conducted test-retest reliability analysis for PA measures (34, 35, 37–39) while only two studies conducted test-retest reliability analysis for SB measures (34, 35). Only three studies performed validity analysis for PA measures (36, 38, 39). No studies assess the criterion validity for SB measures in the included studies. Concerning the statistical method for test-retest reliability and criterion validity, intraclass correlation coefficient and Spearman rank correlation were used frequently across the included studies.

**Table 2:** Basic information of the included studies.

**References**	**Sample characteristics**	**Test-retest reliability**				**Criterion validity**			
		**PA**	**SB**	**Intervals**	**Statistics**	**PA**	**SB**	**Standard**	**Statistics**
Ng et al. (37)	*n* = 2,752 Age: 11 yrs, 13 yrs, and 15 yrs	Past week MVPA	/	/	ICC	/
Tanaka et al. (36)	*n* = 70 (boys = 33) Mean age: 11.3 yrs		(1) Past week MVPA (2) VPA frequency (3) VPA duration	/	Accelerometer	Spearman rank
Bobakova et al. (35)	*n* = 693 (11 yrs = 362; 15 yrs = 331) Slovakia (*n* = 227) Czech (*n* = 353) Poland (*n* = 113)	(1) Past week MVPA (2) VPA frequency	(1) TV use (weekdays and weekend) (2) Computer use for play (weekdays and weekend) (3) Computer use for other purposes (weekdays and weekend)	1–4 w	ICC	/
Yang et al. (34)	*n* = 95 (11 yrs, 15 yrs)	(1) Past week MVPA (2) Typical week MVPA (3) VPA frequency (4) VPA duration	(1) TV use (weekdays and weekend) (2) Computer use for play (weekdays and weekend) (3) Computer use for other purposes (weekdays and weekend)	3 w	ICC	/
Rangul et al. (38)	*n* = 71 (boys = 31); Mean age: 14.9 yrs (13–18 yrs)	(1) VPA frequency (2) VPA duration	/	8–12 d	ICC	(1) VPA frequency	/	VO_2_max; Energy expenditure; Physical activity level	Spearman rank
						(2) VPA duration			
Booth et al. (39)	Reliability study 8 yrs (*n* = 121) and 10 yrs (*n* = 105); Validity study 8 yrs (*n* = 1,072) and 10 yrs (*n* = 954)	(1) VPA frequency (2) VPA duration	/	2 w	Kappa and Agreeme-nt (%)	(1) VPA frequency	/	Aerobic fitness test	Regression
						(2) VPA duration			

*PA, physical activity; SB, sedentary behavior; MVPA, moderate to vigorous physical activity; VPA, vigorous physical activity; w, week(s); d, day(s); /, not available or not studied*.

Supplementary Table 1 shows the summarized results of coefficients of reliability and validity as well as their evaluated performance of the included studies in this review. In terms of the reliability of PA measures, most included studies reported that coefficient test-retest reliability coefficient ranged from about 0.5 to about 0.8, regardless of PA measurement items and subgroups, which indicated that PA measures showed moderate (or above) test-retest reliability (34, 35, 37, 38). In the two studies reporting the reliability coefficients of SB measures (34, 35) and the coefficients of different SB measures varied greatly (from 0.16 to 0.90; signifying poor to almost perfect) (34, 35). The two studies that reported the validity coefficients of PA measures showed, indicating a fair level of validity performance in PA measures (36, 38).

Table 3 exhibits the methodological quality assessments of the included studies for reliability analysis using the COSMIN tool. The scores of quality assessment varied from 4 to 7. Although four studies had a full score of 11 while another study had a full score of 13, the results of each study's quality assessment were not high.

**Table 3:** Methodological quality assessment for test-retest reliability of the included studies.

**References**	**Item 1**	**Item 2**	**Item 3**	**Item 4**	**Item 5**	**Item 6**	**Item 7**	**Item 8**	**Item 9**	**Item 10**	**Item 11**	**Item 12**	**Item 13**	**Item 14**	**Total score**
Ng et al. (37)	No	No	Yes	No	No	No	Yes	No	No	No	Yes	/	/	/	5^*^
Bobakova et al. (35)	No	No	Yes	No	Yes	Yes	Yes	Yes	Yes	No	Yes	/	/	/	8^*^
Yang et al. (34)	No	No	No	No	Yes	Yes	Yes	Yes	Yes	No	Yes	/	/	/	7^*^
Rangul et al. (38)	No	No	No	No	Yes	Yes	Yes	No	Yes	No	Yes	/	/	/	6^*^
Booth et al. (39)	No	No	Yes	No	Yes	Yes	Yes	Yes	Yes	No	/	Yes	No	No	8^&^

*/, not available in this study; item 10 is negative counting (No = 1 score)*;

* *full score is 11*;

& *full score is 13*.

Table 4 displays the methodological quality assessments of the included studies for validity analysis using the COSMIN tool. The three studies that conducted validity analysis all gained 2 scores on quality assessment, indicating low quality.

**Table 4:** Methodological quality assessment for validity of the included studies.

**References**	**Item 1**	**Item 2**	**Item 3**	**Item 4**	**Item 5**	**Item 6**	**Item 7**	**Total score**
Tanaka et al. (36)	No	No	No	Yes	No	Yes	/	3^*^
Rangul et al. (38)	No	No	No	Yes	No	Yes	/	3^*^
Booth et al. (39)	No	No	Yes	No	No	Yes	/	3^*^

*/, not available; item 5 is negative counting (No = 1 score)*;

* *full score is 6; COSMIN (40): consensus-based standards for the selection of health measurement instruments*.

## Discussions

This comprehensive review summarized the evidence on the reliability and validity of PA and SB assessments derived from the HBSC questionnaire. This review also assessed the methodological quality of each study included that conducted reliability or validity analysis using the COSMIN tool (40). This systematic review had some research findings as follows. First, we found that only a few studies have examined the reliability and validity of PA and SB measures derived from the HBSC questionnaire. Second, the reliability of PA measures showed an acceptable level across the included studies while the validity of PA measures presented a fair level. Third, the reliability of SB measures showed a great variation in the performance while no studies assess the validity of SB measures. Fourth, the quality assessment revealed that studies that conducted reliability and validity of PA and SB measures derived from the HBSC questionnaire all showed a low quality, which casts doubts on those studies' results and findings.

PA and SB measures of the HBSC questionnaires have been used in many national surveys, such as in China (47–49) and some European countries (24, 50, 51). However, this review revealed that only a few studies have examined the properties of these measurements in particular populations (34–39). The limited number (*n* = 6) of targeted studies indicated that these PA and SB measures have limited feasibility and utility in other young populations. On this standing, more studies in the future are encouraged to examine the reliability and validity of the PA and SB measures because adequate and vigorous validation on PA and SB measures derived from the HBSC questionnaire is an essential foundation for large-scale use (34, 36). With more studies on the reliability and validity of PA and SB measures of the HBSC questionnaires, its adaptability can be enlarged into different cultures, countries, and societies (39).

Another interesting finding in addition to a few studies that conducted reliability and validity assessments is that some age groups were missing from the reliability study. For example, in Yang et al. (34), the authors' study failed to examine the reliability of PA measures in adolescents aged 13 years. In Ng et al. (37), they did not include adolescents aged 12–14 years. Such issues also occurred in other studies (35, 39). Thus, theoretically, the current evidence can only inform the PA measures had satisfactory reliability in some particular adolescents with specific ages instead of all the adolescent populations. We thereby advocate that more studies should address this issue to expand the generalizability that PA measures are reliable for adolescents with a wider age range.

This review found that PA measures of the HBSC questionnaire show an acceptable test-retest reliability. This in turn indicates that the PA measures of the HBSC questionnaire are a reliable measure to collect PA data or information in adolescent populations. Interestingly, only one study by Yang et al. (34) examined the reliability of PA over the usual week and this study showed that this question for PA measure had satisfactory reliability in Chinese samples (Beijing). However, the current evidence is insufficient to support this kind of PA measure having good reliability. It is therefore urgently needed to examine the reliability of measurement of usual weekly PA by more studies in the future.

Concerning the reliability of SB measures, only two studies reported the coefficient values (34, 35), which indicated that different questions of SB measures had varying coefficients of reliability across different subpopulations. For example, in Polish samples, the questions of sitting measures had coefficients over 0.9 of reliability, indicating a perfect performance (35). However, those measures showed a poor performance in the Chinese samples of 15 years in Yang et al.'s study (34). Such a large inconsistency may be owing to different measurement protocols, sociocultural country differences (34, 35). However, overall, the SB measures of the HBSC questionnaire showed moderate (acceptable) reliability regardless of sex, age, and national difference. This suggests that SB measures of HBSC are reliable in capturing information on SB among adolescents. We still recommend that more studies should re-examine the reliability of SB measures of the HBSC questionnaire in more young populations.

There were two included studies in this review that examined the validity of PA measures of the HBSC questionnaire (36, 39), demonstrating fair to moderate performance in validity. This evidence could illustrate that PA measures of the HBSC questionnaire are partially valid in assessing young people's PA. However, only two studies examining the validity of PA measures are inadequate to inform any robust conclusion that PA measures of the HBSC questionnaire are valid when assessing PA in younger populations with different socio-cultural backgrounds. More studies are encouraged to conduct validity analysis in other young populations.

Surprisingly, no studies so far assessed the validity of SB measures of the HBSC questionnaire in the current review. It is therefore acknowledged that the SB measures of the HBSC questionnaire may not be valid in assessing SB among adolescents. We also have to admit that assessing SB is a complex scientific issue (15, 52). However, because SB measures of the HBSC have been used frequently in many national surveillances, knowing the validity of SB measures is a vital foundation to estimate SB more accurately. Thus, addressing this research gap would be greatly beneficial to increase the use of SB measures of the HBSC questionnaire across the world. To achieve these research aims, well-designed measurement protocols are strongly recommended in the future.

This systematic review assessed the study quality of the included studies, which found that the included studies had quality shortcomings when conducting test-retest reliability and validity. For the studies that conducted test-retest reliability, there were some methodological issues. For example, according to the COSMIN guidelines, some studies did not include sufficient sample size (recommended sample size = 100) to perform the test-retest reliability analysis (34, 38). One study by Ng et al. even failed to report the interval days for test-retest reliability (37). Similar quality weaknesses of the included studies that conducted validity analysis were also observed. For example, Booth et al.'s (39) study used an aerobic fitness test to examine the validity of PA measures. However, it is well-known that the aerobic fitness test can be viewed as a goal-standard for PA measures validity examination. In addition, there were research issues involving sample size for validity study (36, 38). In this regard, it is noticeable that previous studies that conducted reliability or validity analysis for PA and SB measures of the HBSC questionnaire had some inherent study design shortcomings, which may negatively influence the interpretations of the results of the studies. It is strongly recommended that future studies should undertake more standardized and rigorous study design to examine the reliability and validity of PA and SB measures of the HBSC questionnaire.

### Study Strengths and Limitations

A primary strength of this review is that we firstly assessed literature for evidence on reliability and validity of PA and SB measurements derived from the HBSC questionnaire. This study highlights the challenges of using the HBSC questionnaires in many populational surveillance surveys across the world. Second, concerning the studies that examine the reliability and validity of PA and SB measures of the HBSC questionnaire, this review is first to assess the study quality, which can identify research gaps for future similar studies. Third, this study provides strong evidence of the validity and reliability of PA and SB items from the HBSC questionnaire, standardizing the use of the questionnaire in future research. However, one study limitation should be mentioned in our review. This limitation is that the literature search and included studies are restricted in English, which may omit some studies published in other languages.

## Conclusions and Recommendations

This study offers systematic evidence on the reliability and validity of the HBSC questionnaire (selected items) in assessing PA and SB among young populations across the world. This systematic review study indicates that PA and SB measures of the HBSC questionnaire are reliable (moderate agreement) in assessing PA and SB among adolescents. However, when assessing PA, the PA measures show fair to moderate performance, indicating being partially valid. The validity of SB measures remains unknown, which should be filled by future research.

Based on the present review study, it is highly recommended that more studies should re-examine the reliability and validity of the PA and SB measures of the HBSC questionnaire in more young populations using a more standardized study design. By doing this, the PA and SB measures of the HBSC questionnaire can be used for health surveillance in a wider range of populations in the world.

## Data Availability Statement

The raw data supporting the conclusions of this article will be made available by the authors, without undue reservation.

## Author Contributions

YS and HW contributed to the conception and design of the study. S-TC and J-TH performed the literature search. YS, YZ, and HW conducted data extraction and quality assessment. HW oversaw the literature search and data analysis and supported the development of the original draft. YS wrote the first draft of the manuscript. YZ, S-TC, and J-TH wrote and edited the Introduction, Methods, and Results sections. All authors contributed to manuscript revision, read, and approved the submitted version.

## Conflict of Interest

The authors declare that the research was conducted in the absence of any commercial or financial relationships that could be construed as a potential conflict of interest.

## Publisher's Note

All claims expressed in this article are solely those of the authors and do not necessarily represent those of their affiliated organizations, or those of the publisher, the editors and the reviewers. Any product that may be evaluated in this article, or claim that may be made by its manufacturer, is not guaranteed or endorsed by the publisher.
