# Supplementary material for: Is the Health Behavior in School-Aged Survey Questionnaire Reliable and Valid in Assessing Physical Activity and Sedentary Behavior in Young Populations? A Systematic Review
Source: Front Public Health. 2022 Mar 28;10:729641. doi: 10.3389/fpubh.2022.729641 (PMC8995780; doi:10.3389/fpubh.2022.729641)
Supplement: Supplementary file 1 [file Table_1.docx]

**Supplementary Table 1. Performance of reliability and validity of each included study**

| **Study/year** |  | **Test-retest reliability** | |  | **Criterion validity** | |
| --- | --- | --- | --- | --- | --- | --- |
|  |  | **Coefficient** | **Performance** |  | **Coefficient** | **Performance** |
| Ng et al. (2019) [37] |  | **Past week MVPA ICC**  Total: 0.720;  15 yrs:  Boys: 0.765  Girls: 0.743  13 yrs:  Boys: 0.694  Girls: 0.745  11 yrs:  Boys: 0.565  Girls: 0.665 | **Past week MVPA ICC**  Total: substantial  15 yrs:  Boys: substantial  Girls: substantial  13 yrs:  Boys: substantial  Girls: substantial  11 yrs:  Boys: moderate  Girls: moderate |  | / | |
| Tanaka et al. (2017) [36] |  | / | |  | (1) Past week MVPA = 0.399 (*p* = 0.004)  (2) VPA frequency = 0.515 (*p* < 0.001)  (3) VPA duration = 0.400 (*p* < 0.001) | (1) **Past week MVPA**: fair  (2) **VPA frequency**: moderate  (3) **VPA duration**: fair |
| Bobakova et al. (2015) [35] |  | **Past week MVPA**  All: 0.60  Boys: 0.53  Girls: 0.51  11 yrs: 0.52  15 yrs: 0.52  Slovakia: 0.51  Czech: 0.53  Poland: 0.98  **VPA frequency**  All: 0.62  Boys: 0.56  Girls: 0.53  11 yrs: 0.52  15 yrs: 0.58  Slovakia: 0.62  Czech: 0.49  Poland: 0.90  **SB**  All: 0.54-0.66  Boys: 0.45-0.57  Girls: 0.52-0.67  11 yrs: 0.45-0.59  15 yrs: 0.42-0.52  Slovakia: 0.54-0.62  Czech: 0.47-0.62  Poland: 0.66-0.92 | **Past week MVPA**  All: moderate  Boys: moderate  Girls: moderate  11 yrs: moderate  15 yrs: moderate  Slovakia: moderate  Czech: moderate  Poland: almost perfect  **VPA frequency**  All: substantial  Boys: moderate  Girls: moderate  11 yrs: moderate  15 yrs: moderate  Slovakia: moderate  Czech: moderate  Poland: almost perfect  **SB**  All: moderate to substantial  Boys: moderate  Girls: moderate to substantial  11 yrs: moderate  15 yrs: moderate  Slovakia: moderate to substantial  Czech: moderate to substantial  Poland: substantial to almost perfect |  | / | |
| Liu et al. (2010) [34] |  | **Past week MVPA**  All: 0.82  Boys: 0.79  Girls: 0.87  11 yrs: 0.81  15 yrs: 0.79  **Typical week MVPA**  All: 0.74  Boys: 0.73  Girls: 0.76  11 yrs: 0.77  15 yrs: 0.71  **VPA frequency**  All: 0.68  Boys: 0.61  Girls: 0.73  11 yrs: 0.72  15 yrs: 0.62  **VPA time per week**  All: 0.57  Boys: 0.50  Girls: 0.66  11 yrs: 0.58  15 yrs: 0.56  **SB**  All: 0.33-0.78  Boys: 0.45-0.83  Girls: 0.19-0.91  11 yrs: 0.38-0.86  15 yrs: 0.16-0.79 | **Past week MVPA**  All: almost perfect  Boys: substantial  Girls: almost perfect  11 yrs: almost perfect  15 yrs: substantial  **Typical week MVPA**  All: substantial  Boys: substantial  Girls: substantial  11 yrs: substantial  15 yrs: substantial  **VPA frequency**  All: substantial  Boys: substantial  Girls: substantial  11 yrs: substantial  15 yrs: substantial  **VPA time per week**  All: moderate  Boys: moderate  Girls: substantial  11 yrs: moderate  15 yrs: moderate  **SB**  All: fair to substantial  Boys: moderate to almost perfect  Girls: poor to almost perfect  11 yrs: fair to almost perfect  15 yrs: poor to substantial |  | / | |
| Rangul et al. (2008) [38] |  | **VPA frequency**  All: 0.73  Boys: 0.59  Girls: 0.87  13-15 yrs: 0.71  16-18 yrs: 0.76  **VPA duration**  All: 0.71  Boys: 0.66  Girls: 0.76  13-15 yrs: 0.62  16-18 yrs: 0.85 | **VPA frequency**  All: substantial  Boys: moderate  Girls: almost perfect  13-15 yrs: substantial  16-18 yrs: substantial  **VPA duration**  All: substantial  Boys: substantial  Girls: substantial  13-15 yrs: substantial  16-18 yrs: almost perfect |  | **VPA frequency (against VO_2_max)**  All: 0.39 (*p* < 0.01)  **VPA frequency (against energy expenditure)**  All: not significant  **VPA frequency (against physical activity level)**  All: not significant  **VPA duration (against VO_2_max)**  All: 0.33 (*p* < 0.01)  **VPA duration (against energy expenditure)**  All: not significant  **VPA duration (against physical activity level)**  All: not significant | **VPA frequency (against VO_2_max)**  All: fair  **VPA duration (against VO_2_max)**  All: fair |
| Booth et al. (2001) [39] |  | **VPA frequency**  Year 8  Boys:  K = 0.36 (0.20-0.51)  A = 41% (29-54)  Girls:  K = 0.44 (0.25-0.64)  A = 56% (42-69)  Year 10  Boys:  K = 0.60 (0.48-0.72)  A = 57% (45-69)  Girls:  K = 0.57 (0.34-0.80)  A = 59% (39-77)  **VPA duration**  Year 8  Boys:  K = 0.22 (0.05-0.39)  A = 35% (23-48)  Girls:  K = 0.26 (0.09-0.44)  A = 28% (17-41)  Year 10  Boys:  K = 0.58 (0.47-0.70)  A = 44% (33.56)  Girls:  K = 0.52 (0.29-0.75)  A = 50% (31-69) | **VPA frequency**  Year 8  Boys: fair  Girls: moderate  Year 10  Boys: moderate  Girls: moderate  **VPA duration**  Year 8  Boys: fair  Girls: fair  Year 10  Boys: moderate  Girls: moderate |  | **VPA frequency and duration**  Participants with more VPA frequency and durations showed better levels of aerobic fitness regardless of year and sex. | **VPA frequency and duration**  Cannot be evaluated qualitatively according to the results |
